# A cryogenic spin-torque memory element with precessional magnetization dynamics

**DOI:** 10.1038/s41598-018-37204-3

**Published:** 2019-01-28

**Authors:** G. E. Rowlands, C. A. Ryan, L. Ye, L. Rehm, D. Pinna, A. D. Kent, T. A. Ohki

**Affiliations:** 1 Raytheon BBN Technologies, Cambridge, MA 02138 USA; 2 Center for Quantum Phenomena, Department of Physics, New York University, New York, NY 10003 USA

## Abstract

We present a study of precessional magnetization switching in orthogonal spin-torque spin-valve devices at low temperatures. The samples consist of a spin-polarizing layer that is magnetized out-of-the film plane and an in-plane magnetized free and reference magnetic layer separated by non-magnetic metallic layers. We find coherent oscillations in the switching probability, characterized by high speed switching (~200 ps), error rates as low as 10^−5^ and decoherence effects at longer timescales (~1 ns). Our study, which is conducted over a wide range of parameter space (pulse amplitude and duration) with deep statistics, demonstrates that the switching dynamics are likely dominated by the action of the out-of-plane spin polarization, in contrast to in-plane spin-torque from the reference layer, as has been the case in most previous studies. Our results demonstrate that precessional spin-torque devices are well suited to a cryogenic environment, while at room temperature they have so far not exhibited coherent or reliable switching.

## Introduction

Spin-transfer devices that operate at low temperature are of interest for applications that require a cryogenic memory, such as Josephson junction based logic circuits^1^. A requirement for this application is high-speed operation with relatively low energy dissipation. Conventional spin-transfer torque (STT) devices, such as those being developed as commercial room temperature memories, typically rely on thermal fluctuations to instigate switching and thus may not be suitable for low temperature operation. A conventional STT device consists of two thin magnetic layers separated by a non-magnetic layer, with the memory states being the layer magnetizations aligned either parallel or antiparallel. However, the initial spin-transfer torque in these states is small and only increases once a fortuitous thermal field causes a deflection of the magnetization, leading to nanosecond incubation delays for switching and stochastic switching characteristics^2–4^. At low temperatures, the further reduction in the magnitudes of thermal fluctuations can lead to increase delays without engineering any additional source of initial torque.

An orthogonal spin-transfer (OST) device overcomes this limitation by having a spin-polarizing layer aligned perpendicular to the free layer that provides a large spin-torque the moment a current is applied^5^. This perpendicular polarizer induces precessional magnetization dynamics, as it forces the free layer magnetization out of the film plane leading to coherent precessional motion of the magnetization about the film normal: the magnet’s hard magnetic axis^6^. Macrospin simulations show that, for a sufficiently strong out of plane spin polarization, the magnetization should exhibit several full scale probability oscillations as a function of the applied current pulse duration^7^. While experiments at room temperature^7–13^ and cryogenic temperatures^14^ have demonstrated fast (0.1–1.0 ns) switching, these macrospin predictions have not been borne out. In those studies showing evidence of probability oscillations, decoherence appears to suppress the switching probabilities beyond a single oscillation^7,14^. In one case multiple oscillations are observed, but switching probabilities never approach 100%, possibly as a result of thermal decoherence and vortex formation in the free layer^15^. Furthermore, these experiments were conducted over a relatively limited region of the phase space of applied pulse currents and durations. The rich phase space predicted in simulations remains relatively unexplored.

Here we study switching in OST spin-valve based devices at *T* = 4 K with an experimental setup designed to minimize thermal noise at the device and increase the overall measurement throughput. This enables us to test a wide range of pulse amplitudes and durations and construct detailed phase diagrams of coherent magnetization dynamics. We demonstrate high speed switching for pulse lengths down to 100 ps and find error rates as low as 10^−5^ that represent a large improvement over room temperature results. Additionally, we perform finite-temperature stochastic Landau-Lifshitz-Gilbert-Slonczewski (LLGS) simulations^16^ as a basis for comparison.

## Results

**Figure 1:**
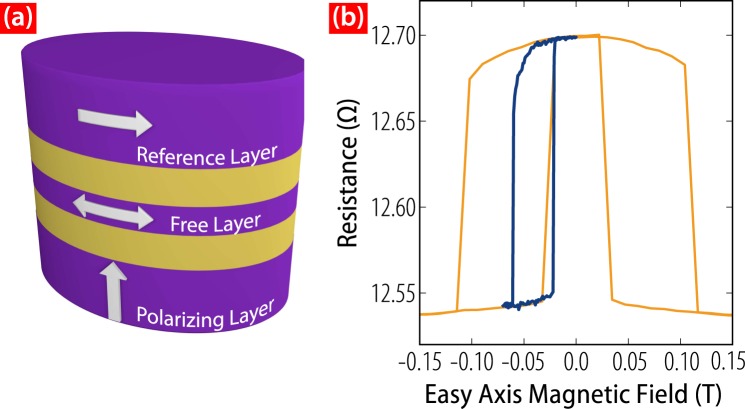
(**a**) Schematic of an OST device, with the equilibrium magnetization directions of the free, fixed, and polarizing layers indicated. (**b**) Major (orange) and minor (blue) hysteresis loops of a device at *T* = 4 K.

The OST devices under study are 50 nm × 100 nm elliptical nanopillars with a CoFeB (3) magnetic free layer (FL), an in-plane reference layer (RL), and an out-of-plane magnetized spin-polarizing layer (PL) as shown in Fig. 1(a). Full stack details are given in the Methods section.

Switching studies are performed with a bias field set in the center of the minor hysteresis loop of Fig. 1(b). These devices operate in a different regime than those from most previous studies, inasmuch as the out-of-plane spin polarization exceeds the in-plane spin polarization. Consequently, the free layer’s magnetization is induced to oscillate out-of-plane by current pulses of either polarity irrespective of whether the system starts in the antiparallel (AP) or parallel (P) configuration. The action of the in-plane polarization does not determine the switching polarity; instead it only modifies the details of the magnetic dynamics.

**Figure 2:**
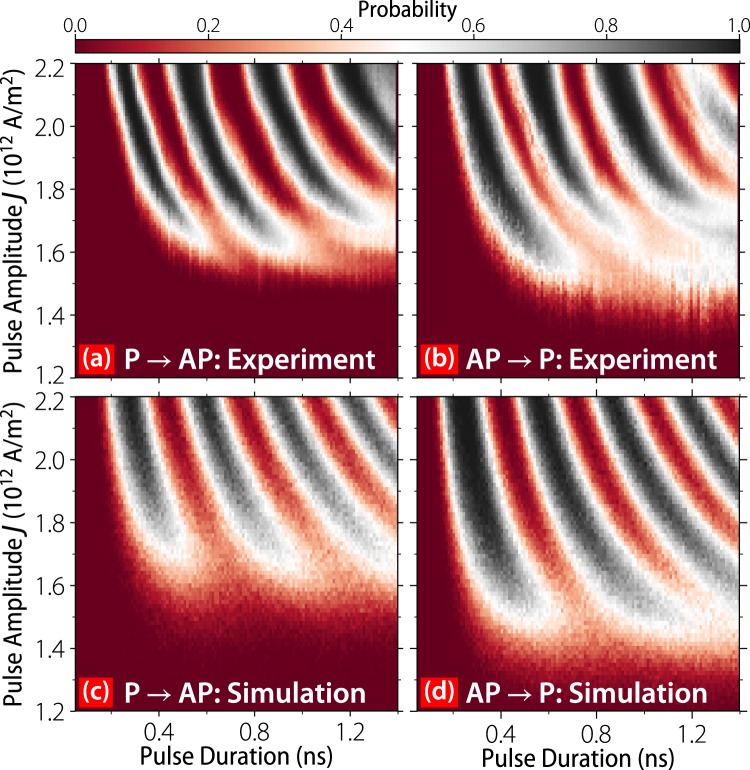
Comparison of *T* = 4 K experimental (**a**,**b**) and *T* = 60 simulated (**c**,**d**) switching phase diagrams for (**a**,**c**) P → AP and (**b**,**d**) AP → P switching polarities. Each pixel represents an estimate of the switching probability from on average 2048 attempts. All data is taken with positive current polarity, which we define as having electron flow from the reference layer to the free layer.

Using the measurement procedure described in the methods section, we build up a phase diagram for precessional switching over a range of pulse amplitudes and durations. These results are shown in Fig. 2(a,b), where each pixel represents the switching probability averaged over ≈2048 switching attempts in each direction (4096 switching attempt for each duration/amplitude pair using the reset scheme described in the methods section). All data is taken with positive currents. In both AP → P and P → AP switching polarities, the sample undergoes three full probability oscillations with a period of approximately 400 ps. For longer pulses the switching probability does not recover to 100%, and for longer pulses yet (not shown) the sample can occasionally become stuck in an intermediate resistance state. Micromagnetic simulations suggest that vortex formation is responsible for this behavior, but that vortices can be disfavored by employing a synthetic antiferromagnet as the perpendicular polarizing layer^15^. The difference between the critical current densities *J*_*c*_ for AP → P and P → AP switching, apparent in Fig. 2(a,b), is a result of the STT from the RL favoring the P state for positive currents and can be counteracted by an applied field, as confirmed in the simulations detailed below.

To better understand these results we perform finite temperature simulations using the macrospin model described in the methods section. We simulate the entire amplitude-duration phase diagram, shown in Fig. 2(c,d), where each pixel gives the switching probability for an ensemble of 512 macrospins subject to different realizations of the thermal field. By varying the magnetic damping *α*, the spin-torque polarizations ${{\mathscr{P}}}_{{\rm{RL}}/{\rm{PL}}}$ and asymmetries ${{\rm{\Lambda }}}_{{\rm{RL}}/{\rm{PL}}}$ of the RL and PL we are able to reproduce the shapes, periodicities, onset currents, and widths of the probability oscillations. This allows us to identify the disparity between critical current densities *J*_*c*_ for different switching polarities, as well as the slightly increased AP → P switching speed, as stemming from the influence of STT from the in-plane RL^17^. By adjusting the simulated offset field by 0.5–1.0 mT, this offset can be eliminated. For negative pulses (not shown), we find reduced switching probabilities that are indicative of a non-zero Λ_*R*_^14^.

**Figure 3:**
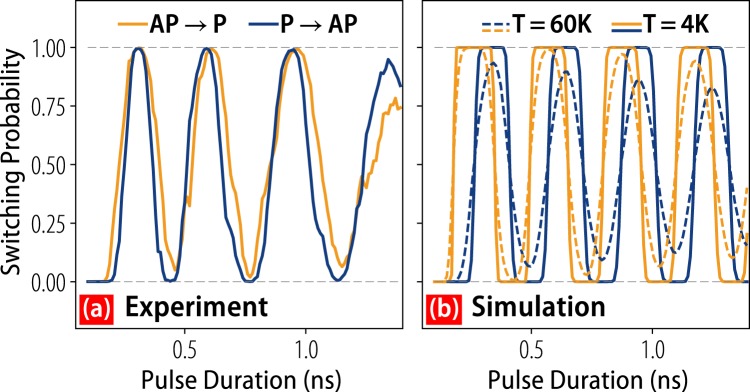
(**a**) Cuts of the experimental (**a**) and simulated (b) phase diagrams taken at *J* = 2.0 × 10^12^A/m^2^. In (**b**) data are shown for *T* = 4 K (solid lines) and *T* = 60 K (dashed lines). AP → P (orange) and P → AP (blue) switching polarities are shown in both (**a**) and (**b**).

The simulations of Fig. 2(c,d) are performed at *T* = 60 K in order to produce a broadening of the switching bands similar to that seen in the experimental data. Estimates of Joule heating in similar structures^18^ suggests a maximum temperature increase of around 30 K, implying that some of the experimentally observed broadening is micromagnetic in nature. Comparing constant pulse-amplitude slices of the simulated and experimental phase diagrams, as shown in Fig. 3(a,b), reveals some further disagreement between model and experiment. At *T* = 4 K the experimental probability oscillations exhibit a sinusoidal behavior before a precipitous decline in probability likely resulting from vortex formation at the sample’s edge^15^. Meanwhile, the simulated *T* = 4 K oscillations exhibit wide high-probability bands with minimal rounding. At *T* = 60 K the simulations show a gradual decoherence of the ensemble resulting from a dephasing along the out-of-plane switching trajectories^19^. Neither simulated behavior is observed in our experimental data, so we infer that both micromagnetic and thermal effects induce broadening and long-timescale decoherence at cryogenic temperatures.

**Figure 4:**
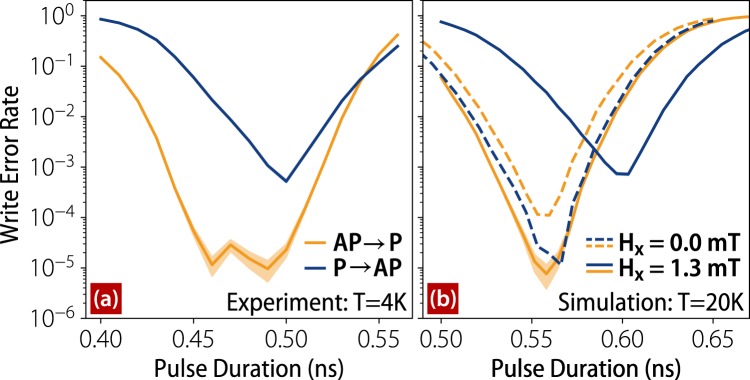
The error rates for AP → P (orange) and P → AP (blue) switching for *J* = 2.2 × 10^12^ A/m^2^ in the vicinity of the second probability oscillation. (**a**) Experimental results at *T* = 4 K and (**b**) simulation results at *T* = 20 K are shown. For (**b**) the dashed lines show the synchronization of the errors rates obtained with an applied easy axis field *H*_*x*_ = 1.3 mT while the solid lines correspond to no applied field. The shaded bands around the solid traces show one standard deviation from the mean error rate as calculated by the beta distribution constructed from the number of successful and failed switching attempts.

We draw attention to the the synchronization of AP → P and P → AP transition probabilities in Fig. 3(a), which are maximized for the same pulse durations unlike in previous experiments. This has important implications for memory write circuitry, which would suffer a substantial increase in complexity were it required to generate pulses of different amplitude or duration. The probabilities can easily be synchronized or desynchronized by the application of in-plane magnetic fields. The low error rates of these devices at low temperatures compel us to perform write error rate (WER) measurements that have not previously been warranted in precessionally switched devices. Near the maximum of the second probability oscillation, as shown in Fig. 4(a), we find that AP → P switching reaches 10^−5^ error rates over a fairly broad window of pulse widths. For P → AP switching, there is a comparatively narrow window where switching reaches a WER of 10^−3^. The error rates for the first oscillation are similar but slightly higher. To our knowledge these errors rates constitute the best performance of a precessionally switched magnetic memory to date. With our accelerated macrospin simulations we run 2^24^ simulations using the same parameters as before and can produce WER behavior shown in Fig. 4(b) assuming a temperature *T* = 20 K that is consistent with expected Joule heating^18^. The imbalance of the WERs for the two polarities is well-captured by simulations and stems from a combination of the external field and STT from the RL (and STT asymmetry thereof). The most reliable operation points can easily be brought together with the application of an external field, as shown in Fig. 4(b). The WERs depend strongly on temperature, and for *T* = 60 K the WER increases to 10^−2^ for AP → P switching. This is expected given that thermal fluctuations are directly responsible for dephasing along the out-of-plane switching trajectories, and suggests that WERs at room temperature will be limited. We emphasize, however, that macrospin simulations can over-estimate the impact of thermal fluctuations on switching behavior since they treat the magnetization as a single rigid domain subject to a single random thermal field.

## Discussion

We have measured high-resolution switching phase diagrams for orthogonal spin-torque devices that exhibit the hallmarks of precessional reversal. For the first time in precessional devices we are able to achieve error rates as low as 10^−5^. However, fine tuning is required before these devices will be practical for usage as a cryogenic main memory.

The inferred spin polarizations *P* in these OST devices appear lower than in similar devices^8,14^. Accordingly the magnetoresistance is suppressed and the critical current densities are elevated. An in depth analysis of how these parameters depend on the deposition conditions is required. Subsequent reduction of the critical current can be achieved by using free layer materials with lower damping^20^, by lowering the magnetization by doping^21^, or by increasing the spin polarization of the magnetic layers^22^. After such modifications the WER should fall well below 10^−6^ given the corresponding reduction in Joule heating.

In terms of practical usage, higher magnetoresistance (~10%) is required before superconducting circuits can be used to quickly read the device’s magnetic state. Above this threshold, state determination can be made in less than 100 ps using a simple two-junction comparator^23^. This low latency is important for read-before-write operations needed for non-deterministic operation (i.e. when the same pulse parameters can switch the device back and forth), as well as for error correction schemes that will bridge the gap between the observed WERs and the <10^−8^ WERs required for practical use.

These devices can also be made to operate in a deterministic mode where the pulse characteristics (i.e. duration or amplitude) determine the final state. More complex write circuitry would be required in this case, but latency would be reduced since read operations would no longer be needed to verify the initial state. Our simulations show that modulation of the easy-axis field and in-plane spin polarization can facilitate deterministic operation: AP → P and P → AP transitions are driven selectively by pulses differing in duration by 200 ps. This behavior is observed in previous experiments, albeit with high error rates^14^.

Using finite temperature macrospin simulations we reproduce many of the qualitative features of the reversal dynamics and identify the distinct influences of STT from both the PL and RL. Macrospin simulations cannot, however, reproduce the sinusoidal probability oscillations without introducing strong decoherence. Micromagnetic simulations suggest that vortex formation may cause the abrupt decrease in switching probabilities, and that such behavior can be mitigated by utilizing a synthetic antiferromagnetic polarizing layer^15^ or thinner magnetic free layers. Despite the computational intensity of finite temperature micromagnetics, such simulations may be required to fully understand the origin of these features.

## Methods

### Devices

The full layer stack of our devices consists of oxidized Si wafer/CuN(60)/Ta(5)/Pd(2)/[Co(0.3)/Pd(0.7)] × 2/[Co(0.15)/Ni(0.6)] × 3/Co(0.15)/Cu(10)/CoFeB(3)/Cu(10)/CoFeB(12)/Cu(10)/Ru(7)/Ta(3). All thicknesses (in parentheses) are given in nm. Nanopillars of various shapes and aspect ratios were fabricated using e-beam lithography and ion-milling of the film stacks described above. The results of this manuscript come from devices with a 50 nm × 100 nm elliptical cross-section, whose FLs have shape anisotropy that defines a magnetic easy axis in the film plane along the long axis of ellipse. Shape anisotropy also sets the magnetization direction of the thicker RL. The major and minor hysteresis loops of one such device are shown in Fig. 1(b), and exhibit a clear offset of the minor loop resulting from an uncompensated dipole field from the RL.

### Experiments

**Figure 5:**
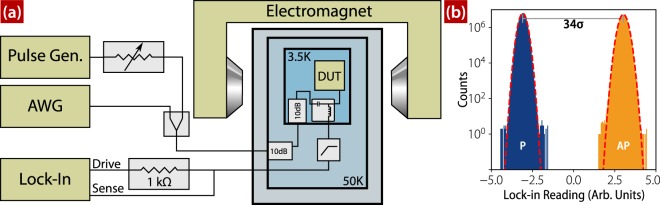
(**a**) Block diagram of the measurement apparatus. High-speed pulses from either the pulse generator or AWG are capacitively coupled into the single-port device under test (DUT) via a bias-T. The low-speed arm of the bias-T is used by the lock-in amplifier to measure the device state. (**b**) Histograms of the sample’s voltage states from a typical run of approximately 110 million shots. The separation is shown in terms of the average standard deviation *σ* of the P and AP states. The red dashed lines are fits to the normal distribution.

Sample chips are mounted and wirebonded in a custom package designed to support microwave signals. The package is mounted on the cold-head of Gifford-McMahon cryocooler (Sumitomo RDK-101D) with a base temperature of ≈4 K when loaded with coaxial lines. Switching pulses are provided by a pulse generator (Picosecond Pulse Labs 10,070 A), and reset pulses by an arbitrary waveform generator (AWG) (Keysight M8190A), both of which are combined and then capacitively coupled to the device via a bias-T (Picosecond Pulse Labs 5575 A) mounted at the cold head. The high speed line has cryogenic attenuators at both the 50 K and 4 K stages to thermalize the center conductor and attenuate thermal noise from higher temperature stages. To provide additional pulse amplitude resolution for the pulse generator (above the 1 dB resolution of the internal step attenuator) we use a voltage controlled variable attenuator (RFMD RFSA2113 evaluation board). The DC coupled port of the bias-T is used to apply current bias and make resistance measurements using a lock-in amplifier (SRS 865) operated at a 1 MHz baseband. The measurement line is low-pass filtered with a custom ECCOSORB low-pass filter^24^ at the 4 K stage again to suppress thermal noise from room temperature. The external magnetic field is applied by a room-temperature electromagnet. A block diagram of the setup is shown in Fig. 5(a).

For each switching attempt we measure the sample’s state before and after applying switching pulses. Because switching occurs independent of polarity and initial state, we select a fixed-polarity reset pulse whose width and amplitude cause the sample to reverse approximately half the time for either initial state. Reset pulses are applied every other attempt, allowing us measure both AP → P and P → AP transitions in the same data run. For a typical data run the initial state distribution using this procedure is 50.9 ± 0.05% P, 49.1 ± 0.05% AP. To maximize the data acquisition rate, the experiment is sequenced by the AWG and is continuously streamed at rate of ≈10 kHz until the desired switching statistics are achieved. A lockin amplifier is used to measure the voltage state of the device with a 1 MHz reference tone, a 10 *μ*s time constant, and a 100 *μ*s settling time corresponding to an 18 dB filter slope. The voltages from the lock-in were clustered into two groups using a k-means algorithm^25^ and are shown in Fig. 5(b). The shape and breadth of the distributions is commensurate with expectations for Johnson noise of the reference resistor as measured with the lockin amplifier settings enumerated above. The P and AP voltage states are well-separated, indicating that there is little chance of misclassifying initial and final resistance states. Occasionally the application of high amplitude switching pulses drove the sample into a metastable intermediate resistance state consistent with a vortex state in the free layer^15^.

The energy delivered to the sample is 235 fJ, as calculated by calibrating the total circuit attenuation and accounting for reflections due to impedance mismatch at the device.

### Simulations

For our simulations the dynamics are described by the LLGS equation:

1 $$\frac{{\rm{d}}{\bf{m}}}{{\rm{d}}\tau }={{\rm{\Gamma }}}_{{\rm{llg}}}+{{\rm{\Gamma }}}_{{\rm{th}}}+{{\rm{\Gamma }}}_{{\rm{stt}}}.$$

as implemented using our open-source, graphics processor unit (GPU) based, macrospin_gpu package^26^. Here **m** = **M**/*M*_*s*_ is the FL magnetization unit vector, Γ_llg_ the deterministic LLG torque, Γ_th_ the thermal torque, and Γ_stt_ the STT. The LLG torque, ${{\rm{\Gamma }}}_{{\rm{llg}}}=-\,{\bf{m}}\times {{\bf{h}}}_{{\rm{eff}}}-\alpha {\bf{m}}\times ({\bf{m}}\times {{\bf{h}}}_{{\rm{eff}}})$, is given in terms of the normalized effective field ${{\bf{h}}}_{{\rm{eff}}}=\frac{-1}{{\mu }_{0}{M}_{s}^{2}V}{\nabla }_{{\bf{m}}}U({\bf{m}})$ for FL volume *V* and damping constant *α*. Time *t* in Eq. 1 has been normalized by the precession frequency so that *τ* = *γμ*_0_*M*_*s*_*t*, where *γ* is the gyromagnetic ratio. The thermal torque Γ_th_ is induced by a Gaussian distributed random field **h**_th_^27^. Although this is a standard assumption we note its applicability to the present regime of cryogenic temperatures and sub-nanosecond switching times is not established. The combined spin-torque contributions from both the out-of-plane PL and in-plane RL can be described in terms of effective spin-polarization vector **n**_stt_:

2 $$\begin{array}{lll}{{\rm{\Gamma }}}_{{\rm{stt}}} & = & \tilde{I}{\bf{m}}\times ({\bf{m}}\times {{\bf{n}}}_{{\rm{stt}}})\\ {{\bf{n}}}_{{\rm{stt}}} & = & {{\mathscr{P}}}_{{\rm{RL}}}\eta ({{\rm{\Lambda }}}_{{\rm{RL}}},{{\bf{m}}}_{x})\hat{{\bf{x}}}+{{\mathscr{P}}}_{{\rm{PL}}}\eta ({{\rm{\Lambda }}}_{{\rm{PL}}},{{\bf{m}}}_{z})\hat{{\bf{z}}}\end{array}$$

3 $$\eta ({\rm{\Lambda }},\,\cos \,\theta )=\frac{2{{\rm{\Lambda }}}^{2}}{({{\rm{\Lambda }}}^{2}+\mathrm{1)}+({{\rm{\Lambda }}}^{2}-\mathrm{1)}\,\cos \,\theta }.$$

Here ${{\mathscr{P}}}_{{\rm{RL}}/{\rm{PL}}}$ and ${{\rm{\Lambda }}}_{{\rm{RL}}/{\rm{PL}}}$ are the spin-torque polarization and asymmetry parameters^28^, respectively, *η*(Λ, cos*θ*) encodes the angular dependence of the spin torque for an angle *θ* between the spin torque polarization and FL, and $\tilde{I}=(\hslash /2e)I/({\mu }_{0}{M}_{s}^{2}V)$ is the normalized applied current.

We take Ms = 1.2 × 10^6^ A/m, *α* = 0.06, ${{\mathscr{P}}}_{{\rm{RL}}/{\rm{PL}}}=0.03/0.05$, ${{\rm{\Lambda }}}_{\mathrm{RL}/\mathrm{PL}}=1.5/1.0$. The shape anisotropy is treated as the combination of two uniaxial contributions: an out-of-plane hard-axis demagnetizing field **h**_d_ = 1 (i.e. the *z* component of the demag tensor *N*_*zz*_ = 1) and an in-plane easy-axis field **h**_an_ = 1 mT. The dipolar field from the reference layer is assumed to be cancelled by the external field and is omitted in our simulations. The ensemble is allowed to thermalize in the AP or P state before a current pulse (rise/fall time of 65/110 ps) is applied.
